# Repetition priming affects guessing not familiarity

**DOI:** 10.1186/1744-9081-3-40

**Published:** 2007-08-14

**Authors:** Richard J Tunney, Gordon Fernie

**Affiliations:** 1 School of Psychology, University of Nottingham, Nottingham, NG7 2RD, UK

## Abstract

**Background:**

The claim that recollection and familiarity based memory processes have distinct retrieval mechanisms is based partly on the observation that masked repetition and semantic priming influence estimates of familiarity derived from *know *responses but have no effect on estimates of recollection derived from *remember *responses. Close inspection of the experiments on which this claim is based reveal the effect size to be small, potentially the result of a type-2 error, and/or inflated due to participants not having the opportunity to report *guesses*. This paper re-evaluates these claims by attempting a partial replication of two such Experiments.

**Methods:**

In Experiment 1 participants made *remember*, *know*, and *guess *responses following primed and unprimed target words. In Experiment 2 participants made *sure*, *unsure*, and *guess *following primed and unprimed target words.

**Results:**

In Experiment 1 the repetition priming effect occurred only for *guess *responses and only for unstudied items. In Experiment 2 the priming effect occurred for both *unsure *and *guess *responses, but again only for unstudied items.

**Conclusion:**

The data are consistent with the view that *remembering *and *knowing *do not correspond to confidence ratings; and suggest that contrary to earlier findings, *recollection *and *familiarity *do not differ in retrieval mechanisms. As such the effects of repetition priming on subjective reports of remembering should not be cited as evidence for the distinction between recollection and familiarity based memory processes.

## Background

Over the past few decades a considerable amount of research has led to the conclusion that recognition memory is composed of at least two functionally distinct processes. These are recollection and familiarity, and this theoretical position has become known as Dual process theory [1]. Proponents of Dual process theory argue that recollection and familiarity give rise to distinct phenomenal states. Recollection-based memory is accompanied by a phenomenal sense of *remembering *in which awareness of the episodic aspects of the study event, such as the context, are consciously re-experienced. By contrast, familiarity-based memory is accompanied by a phenomenal sense of *knowing *whereby the person feels that an item was studied but does not re-experience any specific information about the study event. It follows that the relative contribution of recollection and familiarity can be estimated asking participants to report their subjective experience of remembering by making *remember *and *know *judgments [2,3]. In Tulving's original formulation remembering was thought to be a product of autobiographical memory and knowing associated with semantic memory and conceptual knowledge [3,4]. In recent formulations the distinction between episodic and semantic memory is subsumed by the distinction between recollection and familiarity processes that are more closely associated with neuropsychological systems [5,6]. In each case know responses are treated as a measure of the relative contribution of familiarity based processes and remember responses as a measure of the relative contribution of recollection based memory. That is variations in the conscious experience of remembering provide a method to examine and indeed measure underlying memory processes.

Evidence that recollection and familiarity are functionally distinct, perhaps even independent, processes comes from a number of functional and task dissociations of *remember *and *know *judgments [7]. Some manipulations result in larger estimates of recollection; for example, 'deeper' levels of processing at study result in a higher proportion of *remember *responses relative to 'shallower' levels of processing [2]. Other manipulations result in larger estimates of familiarity, for example non-word recognition is associated with a higher proportion of *know *responses than is word recognition [8].

According to most formulations of Dual process theory recollection and familiarity have distinct retrieval characteristics [1]. According to this account familiarity involves signal detection. To-be-recognized items vary in memory 'strength'; items that have recently been studied have a higher memory 'strength' than items that have not been studied. To decide whether a to-be-recognized item has been studied the participant sets a criterion. Any items that exceed the criterion in memory 'strength' are endorsed as old, while items lower in 'strength' than the criterion are rejected as new. By contrast recollection is thought to involve an all-or-none retrieval mechanism, in the sense that the subject either remembers studying the item (due perhaps to the automatic retrieval of contextual details) or they do not. This model makes two key predictions: First, familiarity is similar to, and should vary with, confidence while recollection should not; and second, familiarity, but not recollection, should be subject to fluency manipulations. That is, any variable that increases an item's memory 'strength' should increase estimates of familiarity but should have no effect on estimates of recollection [9].

A simple and robust method of artificially enhancing processing fluency at test is repetition priming. In this preparation the to-be-recognized target items are preceded by a pattern masked prime that is either the same as (related) or different than (unrelated) the to-be-recognized target. Such repetition priming is known to increase the endorsement rates across both old and new test items [10], but relatively few studies have examined whether artificial manipulations of processing fluency influence confidence ratings and subjective reports of remembering. One such study, reported by Rajaram [11], has become highly influential in the Dual Process literature and appears in the majority of reviews. At the time of writing this study has received 300 citations (Web of Science, accessed Wednesday 21^st ^February 2007). In one experiment Rajaram presented participants with old and new words, half the items were primed by a brief presentation of the target item and half were unprimed (these were preceded by a brief presentation of an irrelevant item). Participants made more *know *judgments to primed than to unprimed items but there was no such effect on the proportion of *remember *judgments. These data are consistent with the view that *know *judgments are based on an assessment of memory 'strength' but *remember *judgments are not. By contrast, when, in a second experiment *remember *and *know *judgments were substituted with categorical confidence ratings of *sure *and *unsure *the repetition priming effect was apparent for both responses. These data suggest that confidence ratings reflect an assessment of memory 'strength' and that *sure *responses do not correspond to *remember *judgments. The important findings of these two experiments is that estimates of recollection based processing based on *remember *judgments are not affected by manipulations of memory 'strength', the corollary being that recollection and familiarity have distinct retrieval mechanisms. However, close inspection of the results of these two experiments reveal issues that, given the influential nature of the study, warrant further analyses and replication.

The first issue concerns the effect sizes. The overall effect size for the repetition priming effect on *know *responses is small, being in the region of Cohen's *d *= .22. Second, the results were analysed by means of a series of pair-wise comparisons that were not corrected for error inflation. The reason behind this was that there had been some disagreement as to whether it is proper to analyse responses of this kind as a factor in Analyses of Variance. Some authors argue that because the responses are mutually exclusive and not independent, ANOVAis inappropriate. Another group argues that ANOVA violating this assumption is at worst trivial and usually inconsequential. However, a problem only occurs if the data iscompositional in nature. Nonetheless Rajaram used planned t-tests rather than an ANOVA to determine whether the tests were justified. The advantage of the ANOVA method is that the comparisons are performed using the overall error term as a control for error inflation. Omitting the ANOVA eliminates this control and is thus subject to error inflation irrespective of whether the tests were planned or otherwise. Examination of the results reported by Rajaram indicate that had the probability of a type-2 error been controlled in these tests the effect may not have been observed. With four relevant comparisons and 23 degrees of freedom the critical values of the Bonferroni corrected *t*-test is 2.71 [12]. Of the two relevant *t*-tests reported by Rajaram only the effect of repetition priming on *know *responses to unstudied words reached the criterion for significance. That is, the effect on studied words may be the result not of repetition priming but of type-2 error inflation. Finally, the repetition priming effect on *know *responses for unstudied items, albeit small, could be inflated as a consequence of the limited range of response options available to participants. Later studies have given participants the option of *guessing *in addition to *remembering *and *knowing *[13]. The rationale for this is that if participants are guessing but do not have the option of reporting their response as such then they are forced to report their guess response as *knowing *on the assumption that it is the nearest level of confidence. This would artefactually inflate estimates of familiarity based on *know *responses.

In a similar experiment to Experiment 3 in Rajaram [11], Rajaram and Geraci [14] demonstrated that semantically related primes influenced estimates of familiarity based on *know *responses but, as in the repetition priming experiments had no effect on *remember *responses. With the exception of the nature of the prime-target relation, the design and analyses of this experiment were identical to Rajaram's Experiment 3. The estimated overall effect size for the *know *responses is again relatively small (Cohen's *d *= .28). Of the four pair-wise comparisons, Rajaram and Geraci reported that "significantly more Know responses were assigned to studied targets when the prime was related than when it was unrelated, *t*(71) = 2.69, *SEM *= .02)". This does reach the critical value of the Bonferroni corrected *t*' for four comparisons with 75 degrees of freedom (2.56, as does the effect on unstudied items (*t*(71) = 4.29). However, rather than reflecting a manipulation of fluency these effects could also be due to the absence of a guess category.

The two experiments that follow aim to re-evaluate the effects of repetition priming on subjective reports of remembering and of confidence. If the effects observed by Rajaram in her Experiment 3 are due to distinct retrieval mechanisms for recollection and familiarity then the inclusion of a *guess *response option should not diminish the effect. On the other hand, if the effect is diminished or eliminated then the effects observed by Rajaram are not evidence for separate retrieval mechanisms and question at least one distinguishing feature of recollection and familiarity. In Experiment 1 participants made *remember*, *know *and *guess *responses. Experiment 2 was identical with the exception that participants made *sure, unsure*, and *guess *responses. The comparison is relevant because, whatever the outcome of Experiment 1, any difference in behaviour for confidence responses would still be consistent with the view that *remembering *and *knowing *do not merely reflect levels of confidence, but would place a question mark over the claim that *remember *and *know *responses are dissociated by repetition priming and the nature of the retrieval mechanisms involved in *recollection *and *familiarity*.

## Methods

### Participants

Twenty members of the University of Nottingham community took part in Experiment 1. Seven were male and thirteen were female. Their mean age was 20 years (*SD *= 0.83). Twenty-nine members of the University of Nottingham community took part in Experiment 2. Thirteen were male and sixteen were female. Their mean age was 24 years (*SD *= 1.20).

### Design & Stimuli

Both experiments used a 2 × 2 design with Item (Old vs. New) and Prime (Related vs. Unrelated) as within-subjects factors. The stimuli consisted of 180 English nouns obtained from the MRC Psycholinguistic database [15]. These were between 5 and 7 letters long (mean = 5.92, *SD *= 0.82). Ninety items were designated as Old items and were presented during study and test. The remaining ninety were designated as New items and were only presented during the test. The prime items consisted of half of the Old and New items so that that half the test items were a repetition of the prime item, and a further set of 90 unrelated prime words selected from the MRC database in the same way.

### Procedure

The study phase of each experiment was identical. Participants were instructed to memorize the words presented on the computer screen. Each word appeared for 3 seconds with an inter-trial interval of 1 second. There was a 15 minute retention interval between the end of the study phase and the start of the test phase. During this period participants were given a newspaper to read. Next, the participants were informed that they would see some more words and to indicate which were old and which were new. Each test trial consisted of four events: first, a mask of 7 ampersands appeared for 500 msecs, followed by the prime word for 50 msecs, and a second mask for 500 msecs, and finally, the target item. No instructions were given regarding the masked prime. In Experiment 1 participants were instructed to report their experience of remembering using the categories *remember*, *know *or *guess *whenever they indicated that an item was old. We used the 'standard' instructions to describe the difference between remembering and knowing [7]. These use face recognition to describe the differences between recollection and familiarity. For example, recollection is described as when a face is recognized along with contextual details of the meeting such as the name of the person and the topic of conversation. Familiarity is described as when a face is recognized but without any of the accompanying contextual details such as the name. Although these instructions differ from those used by Rajaram the two are very similar and are unlikely to result in any differences in how participants report their experiences of remembering. The testing procedure used in Experiment 2 was identical with the exception that whenever an item was identified as old participants were asked to report how confident they were in their decisions using the options of *sure, unsure *and *guess*, instead of making *remember*, *know*, or *guess *judgments.

## Results

### Experiment 1

**Figure 1:**
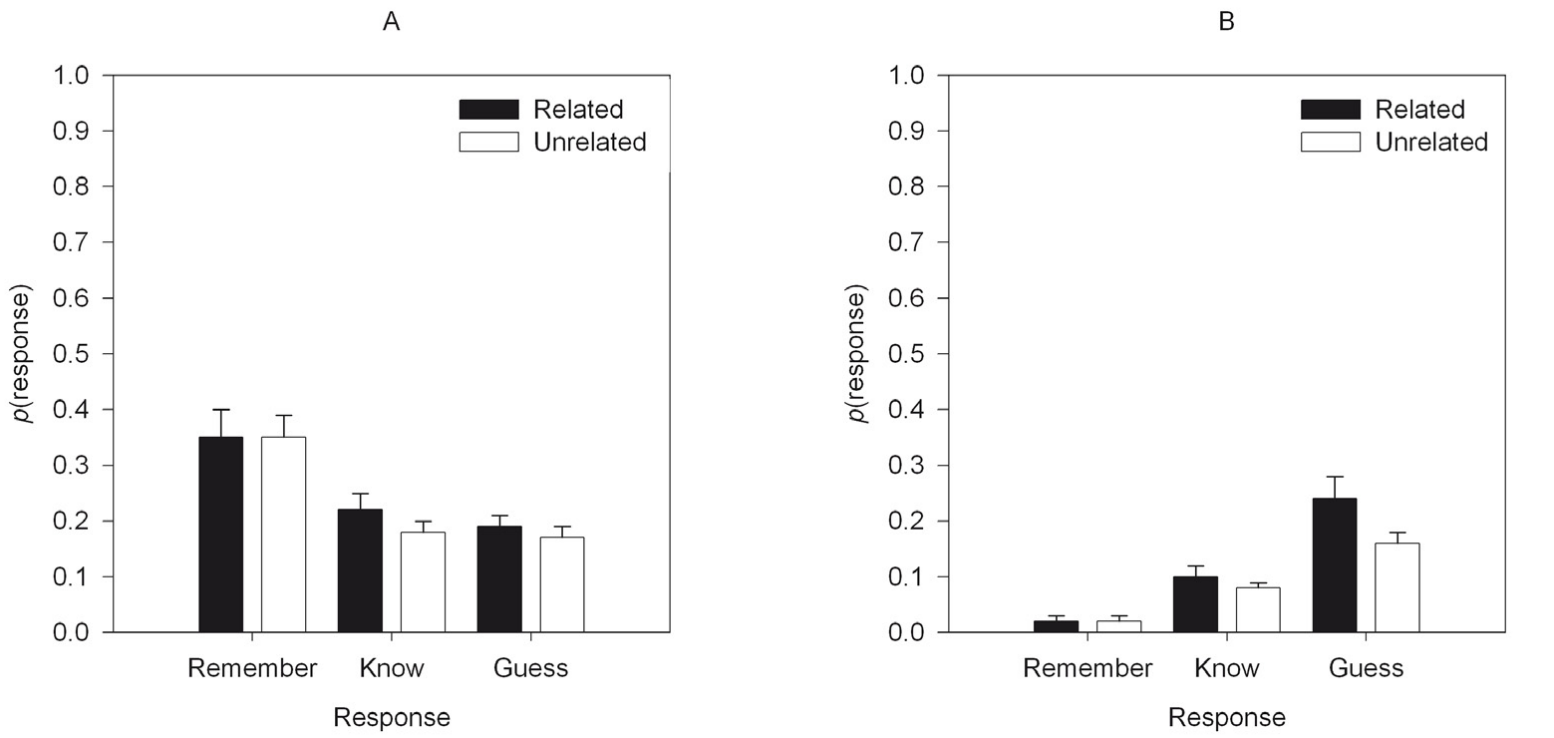
Proportion of *remember*, *know*, and *guess *responses for related and unrelated primes for Panel A: Old items, and Panel B: New Items.

The mean response probabilities for each category of subjective report are shown in Figure 1 as a function of Item and Prime. These data were entered into a 3 × 2 × 2 ANOVA with Response, Item, and Prime as within-subject-factors. The criterion for significance was set to α = .05 for all analyses and degrees of freedom were adjusted using the Greenhouse-Geisser method where the assumption of sphericity was violated. The effect of Response failed to reach significance (*F*(2, 38) = 1.60, *MSE *= 0.03, *p *= .22, ηp2
 MathType@MTEF@5@5@+=feaafiart1ev1aaatCvAUfKttLearuWrP9MDH5MBPbIqV92AaeXatLxBI9gBaebbnrfifHhDYfgasaacH8akY=wiFfYdH8Gipec8Eeeu0xXdbba9frFj0=OqFfea0dXdd9vqai=hGuQ8kuc9pgc9s8qqaq=dirpe0xb9q8qiLsFr0=vr0=vr0dc8meaabaqaciaacaGaaeqabaqabeGadaaakeaaiiGacqWF3oaAdaqhaaWcbaGaeeiCaahabaGaeGOmaidaaaaa@30E5@ = .08). There was an effect of Item (*F*(1, 19) = 25.58, *MSE *< 0.01, *p *< .01, ηp2
 MathType@MTEF@5@5@+=feaafiart1ev1aaatCvAUfKttLearuWrP9MDH5MBPbIqV92AaeXatLxBI9gBaebbnrfifHhDYfgasaacH8akY=wiFfYdH8Gipec8Eeeu0xXdbba9frFj0=OqFfea0dXdd9vqai=hGuQ8kuc9pgc9s8qqaq=dirpe0xb9q8qiLsFr0=vr0=vr0dc8meaabaqaciaacaGaaeqabaqabeGadaaakeaaiiGacqWF3oaAdaqhaaWcbaGaeeiCaahabaGaeGOmaidaaaaa@30E5@ = .87) indicating reliable discrimination between Old and New items; and an effect of Prime (*F*(1, 19) = 22.93, *MSE *< 0.01, *p *< .01, ηp2
 MathType@MTEF@5@5@+=feaafiart1ev1aaatCvAUfKttLearuWrP9MDH5MBPbIqV92AaeXatLxBI9gBaebbnrfifHhDYfgasaacH8akY=wiFfYdH8Gipec8Eeeu0xXdbba9frFj0=OqFfea0dXdd9vqai=hGuQ8kuc9pgc9s8qqaq=dirpe0xb9q8qiLsFr0=vr0=vr0dc8meaabaqaciaacaGaaeqabaqabeGadaaakeaaiiGacqWF3oaAdaqhaaWcbaGaeeiCaahabaGaeGOmaidaaaaa@30E5@ = .55) indicating that Related primes increased endorsements relative to Unrelated primes. There was a two-way interaction between Response and Item (*F*(1.43, 27.11) = 22.06, *MSE *= 0.03, *p *< .01, ηp2
 MathType@MTEF@5@5@+=feaafiart1ev1aaatCvAUfKttLearuWrP9MDH5MBPbIqV92AaeXatLxBI9gBaebbnrfifHhDYfgasaacH8akY=wiFfYdH8Gipec8Eeeu0xXdbba9frFj0=OqFfea0dXdd9vqai=hGuQ8kuc9pgc9s8qqaq=dirpe0xb9q8qiLsFr0=vr0=vr0dc8meaabaqaciaacaGaaeqabaqabeGadaaakeaaiiGacqWF3oaAdaqhaaWcbaGaeeiCaahabaGaeGOmaidaaaaa@30E5@ = .54) that reflects the higher proportion of response of each kind for Old items relative to New ones. The two-way interaction between Response and Prime failed to reach significance (*F*(2, 38) = 1.63, *MSE *< 0.01, *p *= .21, ηp2
 MathType@MTEF@5@5@+=feaafiart1ev1aaatCvAUfKttLearuWrP9MDH5MBPbIqV92AaeXatLxBI9gBaebbnrfifHhDYfgasaacH8akY=wiFfYdH8Gipec8Eeeu0xXdbba9frFj0=OqFfea0dXdd9vqai=hGuQ8kuc9pgc9s8qqaq=dirpe0xb9q8qiLsFr0=vr0=vr0dc8meaabaqaciaacaGaaeqabaqabeGadaaakeaaiiGacqWF3oaAdaqhaaWcbaGaeeiCaahabaGaeGOmaidaaaaa@30E5@ = .08); although there was a reliable interaction between Item and Prime (*F*(1, 19) = 5.62, *MSE *< 0.01, *p *< .03, ηp2
 MathType@MTEF@5@5@+=feaafiart1ev1aaatCvAUfKttLearuWrP9MDH5MBPbIqV92AaeXatLxBI9gBaebbnrfifHhDYfgasaacH8akY=wiFfYdH8Gipec8Eeeu0xXdbba9frFj0=OqFfea0dXdd9vqai=hGuQ8kuc9pgc9s8qqaq=dirpe0xb9q8qiLsFr0=vr0=vr0dc8meaabaqaciaacaGaaeqabaqabeGadaaakeaaiiGacqWF3oaAdaqhaaWcbaGaeeiCaahabaGaeGOmaidaaaaa@30E5@ = .23). The three-way interaction between Response, Item, and Prime failed to reach significance (*F*(2, 38) = 2.44, *MSE *< 0.01, *p *< .10, ηp2
 MathType@MTEF@5@5@+=feaafiart1ev1aaatCvAUfKttLearuWrP9MDH5MBPbIqV92AaeXatLxBI9gBaebbnrfifHhDYfgasaacH8akY=wiFfYdH8Gipec8Eeeu0xXdbba9frFj0=OqFfea0dXdd9vqai=hGuQ8kuc9pgc9s8qqaq=dirpe0xb9q8qiLsFr0=vr0=vr0dc8meaabaqaciaacaGaaeqabaqabeGadaaakeaaiiGacqWF3oaAdaqhaaWcbaGaeeiCaahabaGaeGOmaidaaaaa@30E5@ = .11).

To examine the repetition-priming effects on responses separate analyses were conducted for Old and New items. For the Old items there was a main effect of Response (*F*(1.29, 24.52) = 7.10, *MSE *< 0.05, *p *< .01, ηp2
 MathType@MTEF@5@5@+=feaafiart1ev1aaatCvAUfKttLearuWrP9MDH5MBPbIqV92AaeXatLxBI9gBaebbnrfifHhDYfgasaacH8akY=wiFfYdH8Gipec8Eeeu0xXdbba9frFj0=OqFfea0dXdd9vqai=hGuQ8kuc9pgc9s8qqaq=dirpe0xb9q8qiLsFr0=vr0=vr0dc8meaabaqaciaacaGaaeqabaqabeGadaaakeaaiiGacqWF3oaAdaqhaaWcbaGaeeiCaahabaGaeGOmaidaaaaa@30E5@ = .27) indicating that participants made more *remember *responses than *know *or *guess *responses, and an effect of Prime indicating that items preceded by a Related prime were more likely to be endorsed than those preceded by an Unrelated prime (*F*(1, 19) = 11.02, *MSE *< 0.01, *p *< .01, ηp2
 MathType@MTEF@5@5@+=feaafiart1ev1aaatCvAUfKttLearuWrP9MDH5MBPbIqV92AaeXatLxBI9gBaebbnrfifHhDYfgasaacH8akY=wiFfYdH8Gipec8Eeeu0xXdbba9frFj0=OqFfea0dXdd9vqai=hGuQ8kuc9pgc9s8qqaq=dirpe0xb9q8qiLsFr0=vr0=vr0dc8meaabaqaciaacaGaaeqabaqabeGadaaakeaaiiGacqWF3oaAdaqhaaWcbaGaeeiCaahabaGaeGOmaidaaaaa@30E5@ = .37), but critically there was no interaction between the two (*F*(2, 38) < 1.0, *MSE *< 0.01, *p *= .60, ηp2
 MathType@MTEF@5@5@+=feaafiart1ev1aaatCvAUfKttLearuWrP9MDH5MBPbIqV92AaeXatLxBI9gBaebbnrfifHhDYfgasaacH8akY=wiFfYdH8Gipec8Eeeu0xXdbba9frFj0=OqFfea0dXdd9vqai=hGuQ8kuc9pgc9s8qqaq=dirpe0xb9q8qiLsFr0=vr0=vr0dc8meaabaqaciaacaGaaeqabaqabeGadaaakeaaiiGacqWF3oaAdaqhaaWcbaGaeeiCaahabaGaeGOmaidaaaaa@30E5@ = .03). For the New items there was an effect of Response (*F*(1.27, 24.22) = 29.13, *MSE *< 0.02, *p *< .01, ηp2
 MathType@MTEF@5@5@+=feaafiart1ev1aaatCvAUfKttLearuWrP9MDH5MBPbIqV92AaeXatLxBI9gBaebbnrfifHhDYfgasaacH8akY=wiFfYdH8Gipec8Eeeu0xXdbba9frFj0=OqFfea0dXdd9vqai=hGuQ8kuc9pgc9s8qqaq=dirpe0xb9q8qiLsFr0=vr0=vr0dc8meaabaqaciaacaGaaeqabaqabeGadaaakeaaiiGacqWF3oaAdaqhaaWcbaGaeeiCaahabaGaeGOmaidaaaaa@30E5@ = .61), an effect of Prime (*F*(1, 19) = 19.23, *MSE *< 0.01, *p *< .01, ηp2
 MathType@MTEF@5@5@+=feaafiart1ev1aaatCvAUfKttLearuWrP9MDH5MBPbIqV92AaeXatLxBI9gBaebbnrfifHhDYfgasaacH8akY=wiFfYdH8Gipec8Eeeu0xXdbba9frFj0=OqFfea0dXdd9vqai=hGuQ8kuc9pgc9s8qqaq=dirpe0xb9q8qiLsFr0=vr0=vr0dc8meaabaqaciaacaGaaeqabaqabeGadaaakeaaiiGacqWF3oaAdaqhaaWcbaGaeeiCaahabaGaeGOmaidaaaaa@30E5@ = .50), and an interaction between the two (*F*(1.36, 25.76) = 4.65, *MSE *< 0.01, *p *= .03, ηp2
 MathType@MTEF@5@5@+=feaafiart1ev1aaatCvAUfKttLearuWrP9MDH5MBPbIqV92AaeXatLxBI9gBaebbnrfifHhDYfgasaacH8akY=wiFfYdH8Gipec8Eeeu0xXdbba9frFj0=OqFfea0dXdd9vqai=hGuQ8kuc9pgc9s8qqaq=dirpe0xb9q8qiLsFr0=vr0=vr0dc8meaabaqaciaacaGaaeqabaqabeGadaaakeaaiiGacqWF3oaAdaqhaaWcbaGaeeiCaahabaGaeGOmaidaaaaa@30E5@ = .20).

Multiple pair-wise comparisons using the Bonferroni correction were conducted to examine the effect of Prime on Response for the New items. Rajaram reported that priming increased the proportion of *know *responses, but had no effect on *remember *responses. The same comparisons performed on these data, with the addition of the *guess *response option, reveal no effect on *remember *responses (*t'*(19) = 1.14, *SD *= 0.03, *p *> .05); nor, in contrast to the data reported by Rajaram, on *know *responses (*t*'(19) = 1.53, *SD *= 0.06 *p *> .05). There was however, a reliable effect of priming on *guess *responses (*t*'(19) = 3.16, *SD *= 0.11, *p *< .05). The effect of priming is localized within the *guess *responses to New items, not on familiarity. This effect is not trivial: Cohen's *d *= .71.

In short, the results of this experiment show that the locus of the repetition priming effect on subjective reports of confidence is on *guess *responses to unstudied items. The contention is that effects observed by Rajaram (Experiment 3) are due to (a) type-2 error, and (b) participants reported their *guesses *as *know *responses. Experiment 2 is identical with the exception that participants made *sure *and *unsure *responses instead of *remember *and *know *responses to check that subjective reports of remembering do not correspond to confidence ratings.

### Experiment 2

**Figure 2:**
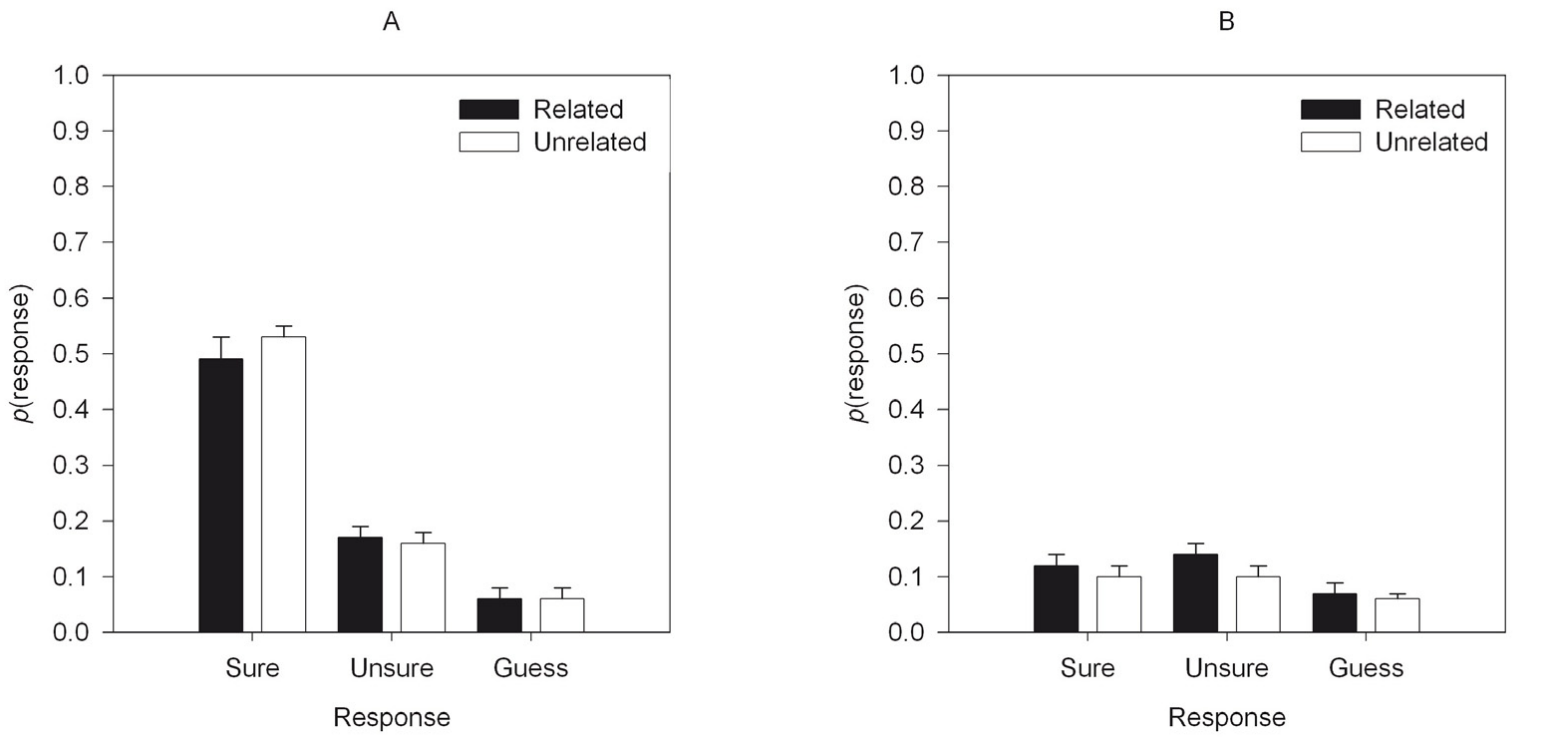
Proportion of *sure*, *unsure*, and *guess *responses for related and unrelated primes for Panel A: Old items, and Panel B: New Items.

The mean response probabilities for each category of subjective report are shown in Figure 2 as a function of Item and Prime. These data were entered into a 3 × 2 × 2 ANOVA with Response, Item, and Prime as within-subject-factors. There was an effect of Response indicating that participants made more *sure *responses than any other (*F*(1.50, 42.02) = 31.32, *MSE *= 0.08, *p *< .01, ηp2
 MathType@MTEF@5@5@+=feaafiart1ev1aaatCvAUfKttLearuWrP9MDH5MBPbIqV92AaeXatLxBI9gBaebbnrfifHhDYfgasaacH8akY=wiFfYdH8Gipec8Eeeu0xXdbba9frFj0=OqFfea0dXdd9vqai=hGuQ8kuc9pgc9s8qqaq=dirpe0xb9q8qiLsFr0=vr0=vr0dc8meaabaqaciaacaGaaeqabaqabeGadaaakeaaiiGacqWF3oaAdaqhaaWcbaGaeeiCaahabaGaeGOmaidaaaaa@30E5@ = .53), and an effect of Item indicating reliable discrimination between Old and New items (*F*(1, 28) = 193.92, *MSE *= 0.01, *p *< .01, ηp2
 MathType@MTEF@5@5@+=feaafiart1ev1aaatCvAUfKttLearuWrP9MDH5MBPbIqV92AaeXatLxBI9gBaebbnrfifHhDYfgasaacH8akY=wiFfYdH8Gipec8Eeeu0xXdbba9frFj0=OqFfea0dXdd9vqai=hGuQ8kuc9pgc9s8qqaq=dirpe0xb9q8qiLsFr0=vr0=vr0dc8meaabaqaciaacaGaaeqabaqabeGadaaakeaaiiGacqWF3oaAdaqhaaWcbaGaeeiCaahabaGaeGOmaidaaaaa@30E5@ = .87); but in contrast with Experiment 1 there was no effect of Prime (*F*(1, 28) = 1.30, *MSE *= 0.03, *p *= .26, ηp2
 MathType@MTEF@5@5@+=feaafiart1ev1aaatCvAUfKttLearuWrP9MDH5MBPbIqV92AaeXatLxBI9gBaebbnrfifHhDYfgasaacH8akY=wiFfYdH8Gipec8Eeeu0xXdbba9frFj0=OqFfea0dXdd9vqai=hGuQ8kuc9pgc9s8qqaq=dirpe0xb9q8qiLsFr0=vr0=vr0dc8meaabaqaciaacaGaaeqabaqabeGadaaakeaaiiGacqWF3oaAdaqhaaWcbaGaeeiCaahabaGaeGOmaidaaaaa@30E5@ = .04). There was an interaction between Response and Item (*F*(1.25, 34.88) = 99.01, *MSE *= 0.02, *p *< .01, ηp2
 MathType@MTEF@5@5@+=feaafiart1ev1aaatCvAUfKttLearuWrP9MDH5MBPbIqV92AaeXatLxBI9gBaebbnrfifHhDYfgasaacH8akY=wiFfYdH8Gipec8Eeeu0xXdbba9frFj0=OqFfea0dXdd9vqai=hGuQ8kuc9pgc9s8qqaq=dirpe0xb9q8qiLsFr0=vr0=vr0dc8meaabaqaciaacaGaaeqabaqabeGadaaakeaaiiGacqWF3oaAdaqhaaWcbaGaeeiCaahabaGaeGOmaidaaaaa@30E5@ = .78) that reflects the higher proportion of *sure *and *unsure *responses for old items relative to new ones; an interaction between Response and Prime (*F*(1.59, 44.62) = 3.41, *MSE *= 0.01, *p *< .05, ηp2
 MathType@MTEF@5@5@+=feaafiart1ev1aaatCvAUfKttLearuWrP9MDH5MBPbIqV92AaeXatLxBI9gBaebbnrfifHhDYfgasaacH8akY=wiFfYdH8Gipec8Eeeu0xXdbba9frFj0=OqFfea0dXdd9vqai=hGuQ8kuc9pgc9s8qqaq=dirpe0xb9q8qiLsFr0=vr0=vr0dc8meaabaqaciaacaGaaeqabaqabeGadaaakeaaiiGacqWF3oaAdaqhaaWcbaGaeeiCaahabaGaeGOmaidaaaaa@30E5@ = .11), and an interaction between Item and Prime (*F*(1, 28) = 10.40, *MSE *< 0.01, *p *< .01, ηp2
 MathType@MTEF@5@5@+=feaafiart1ev1aaatCvAUfKttLearuWrP9MDH5MBPbIqV92AaeXatLxBI9gBaebbnrfifHhDYfgasaacH8akY=wiFfYdH8Gipec8Eeeu0xXdbba9frFj0=OqFfea0dXdd9vqai=hGuQ8kuc9pgc9s8qqaq=dirpe0xb9q8qiLsFr0=vr0=vr0dc8meaabaqaciaacaGaaeqabaqabeGadaaakeaaiiGacqWF3oaAdaqhaaWcbaGaeeiCaahabaGaeGOmaidaaaaa@30E5@ = .27). Finally, like Experiment 1 the three-way interaction between Response, Item, and Prime failed to reach significance (*F*(2, 56) = 1.02, *MSE *< 0.01, *p *= .37, ηp2
 MathType@MTEF@5@5@+=feaafiart1ev1aaatCvAUfKttLearuWrP9MDH5MBPbIqV92AaeXatLxBI9gBaebbnrfifHhDYfgasaacH8akY=wiFfYdH8Gipec8Eeeu0xXdbba9frFj0=OqFfea0dXdd9vqai=hGuQ8kuc9pgc9s8qqaq=dirpe0xb9q8qiLsFr0=vr0=vr0dc8meaabaqaciaacaGaaeqabaqabeGadaaakeaaiiGacqWF3oaAdaqhaaWcbaGaeeiCaahabaGaeGOmaidaaaaa@30E5@ = .04).

To examine the interactions, the effects of Prime on Response were analyzed for Old and New items separately. For the Old items there was an effect of Response resulting from more *sure *responses than *unsure *or *guess *responses (*F*(1.32, 36.45) = 60.74, *MSE *= 0.08, *p *< .05, *η *= .68), but no effect of Prime (*F*(1, 28) = 2.24, *MSE *< 0.01, *p *> .05, ηp2
 MathType@MTEF@5@5@+=feaafiart1ev1aaatCvAUfKttLearuWrP9MDH5MBPbIqV92AaeXatLxBI9gBaebbnrfifHhDYfgasaacH8akY=wiFfYdH8Gipec8Eeeu0xXdbba9frFj0=OqFfea0dXdd9vqai=hGuQ8kuc9pgc9s8qqaq=dirpe0xb9q8qiLsFr0=vr0=vr0dc8meaabaqaciaacaGaaeqabaqabeGadaaakeaaiiGacqWF3oaAdaqhaaWcbaGaeeiCaahabaGaeGOmaidaaaaa@30E5@ = .07) nor an interaction between the two (*F*(1.47, 41.27) = 2.50, *MSE *< 0.01, *p *> .05, ηp2
 MathType@MTEF@5@5@+=feaafiart1ev1aaatCvAUfKttLearuWrP9MDH5MBPbIqV92AaeXatLxBI9gBaebbnrfifHhDYfgasaacH8akY=wiFfYdH8Gipec8Eeeu0xXdbba9frFj0=OqFfea0dXdd9vqai=hGuQ8kuc9pgc9s8qqaq=dirpe0xb9q8qiLsFr0=vr0=vr0dc8meaabaqaciaacaGaaeqabaqabeGadaaakeaaiiGacqWF3oaAdaqhaaWcbaGaeeiCaahabaGaeGOmaidaaaaa@30E5@ = .08). The absence of a priming effect on responses to Old items replicates the pattern observed in Experiment 1 and also reported by Rajaram. The critical comparisons concern the location of the effect for New items. There was no effect of Response for New items (*F*(2, 56) = 2.55, *MSE *= 0.02, *p *= .09, ηp2
 MathType@MTEF@5@5@+=feaafiart1ev1aaatCvAUfKttLearuWrP9MDH5MBPbIqV92AaeXatLxBI9gBaebbnrfifHhDYfgasaacH8akY=wiFfYdH8Gipec8Eeeu0xXdbba9frFj0=OqFfea0dXdd9vqai=hGuQ8kuc9pgc9s8qqaq=dirpe0xb9q8qiLsFr0=vr0=vr0dc8meaabaqaciaacaGaaeqabaqabeGadaaakeaaiiGacqWF3oaAdaqhaaWcbaGaeeiCaahabaGaeGOmaidaaaaa@30E5@ = .08). There was an effect of Prime (*F*(1, 28) = 8.98, *MSE *< 0.01, *p *< .01, ηp2
 MathType@MTEF@5@5@+=feaafiart1ev1aaatCvAUfKttLearuWrP9MDH5MBPbIqV92AaeXatLxBI9gBaebbnrfifHhDYfgasaacH8akY=wiFfYdH8Gipec8Eeeu0xXdbba9frFj0=OqFfea0dXdd9vqai=hGuQ8kuc9pgc9s8qqaq=dirpe0xb9q8qiLsFr0=vr0=vr0dc8meaabaqaciaacaGaaeqabaqabeGadaaakeaaiiGacqWF3oaAdaqhaaWcbaGaeeiCaahabaGaeGOmaidaaaaa@30E5@ = .24), but no interaction between the two (*F*(2, 56) = 1.17, *MSE *< 0.01, *p *= .32, ηp2
 MathType@MTEF@5@5@+=feaafiart1ev1aaatCvAUfKttLearuWrP9MDH5MBPbIqV92AaeXatLxBI9gBaebbnrfifHhDYfgasaacH8akY=wiFfYdH8Gipec8Eeeu0xXdbba9frFj0=OqFfea0dXdd9vqai=hGuQ8kuc9pgc9s8qqaq=dirpe0xb9q8qiLsFr0=vr0=vr0dc8meaabaqaciaacaGaaeqabaqabeGadaaakeaaiiGacqWF3oaAdaqhaaWcbaGaeeiCaahabaGaeGOmaidaaaaa@30E5@ = .04).

Pair-wise comparisons using the Bonferroni correction were conducted to examine the effect of Prime on Response for New items separately. In Experiment 1 the only effect of repetition priming was on *guess *responses. The same comparisons performed on these data reveal no effect on *sure *responses (*t'*(28) = 1.28, *SD *= 0.06, *p *> .05); there was an effect on *unsure *responses (*t*'(28) = 2.70, *SD *= 0.7, *p *< .05) and a marginal effect on *guess *responses to new items (*t*'(28) = 2.21, *SD *= 0.04, *p *< .05).

The results of Experiment 2 are consistent with the view that subjective reports of remembering do not correspond to subjective reports of confidence. That is, although Experiment 1 places a question mark over whether or not recollection and familiarity differ in retrieval, subjective reports of the experience of remembering remain a useful and valid means to study them.

## Discussion

The effects of repetition priming on subjective reports of remembering and confidence were re-evaluated in two Experiments. The claim that repetition priming increases *know *responses but not *remember *responses has been important in the development of dual process models of recognition memory. The re-evaluation was motivated by close inspection of two experiments on which this claim is partly based [11]. That is, the previously reported effects were small, based on uncorrected pair-wise comparisons, and potentially inflated by the absence of a *guess *response option. Given the influential nature of these studies in our understanding of recognition memory a replication was necessary and justified. Our results showed that the locus of the repetition priming effect on subjective reports of remembering was solely on *guess *responses to previously unstudied items; not, as had previously been reported, on *know *responses to both studied and unstudied items. Experiment 2 also found that the repetition priming effect was restricted to unstudied items, but increased the proportion of both *unsure *and *guess *responses. There was no effect on the proportion of *sure *responses. One possibility is that the use of a 3-point confidence scale may not have been sensitive enough to detect an effect of priming on studied items. However, earlier research suggests that this is unlikely because in related experiments shorter scales (2-point) are more sensitive than longer confidence scales (50% to 100%) [16,17].

## Conclusion

It is important to note that the arguments herein are not based on null results. In each case reliable main effects and interactions were obtained, even when comparisons were adjusted for error inflation. Instead the arguments are based on the finding that the locus of the repetition priming effect is when participants guess about items that they have not seen before. This has an obvious implication for dual process theory. If manipulations of processing fluency do not differentially affect either measure of the subjective experience of remembering, then recollection and familiarity may share the same retrieval mechanism. They may remain distinct and even independent processes, but in this respect they are similar. It is ironic that the increase in *unsure *and *guess *responses as a result of priming observed in Experiment 2 is similar to the pattern previously claimed for *know *responses. However, the fact that the pattern of results differs according to whether participants report their experience of remembering or their confidence in their responses is consistent with the view that *remember *and *know *responses do not merely reflect levels of confidence.

An alternative to questioning the existence of distinct retrieval mechanisms for recollection and familiarity has been to assume that they exist but to attempt to fit a single-process model. Recently Dunn [18] provided a cogent exposition of how the apparent dissociation between recollection and familiarity on the one hand, and confidence on the other, could be more parsimoniously explained by a signal detection model. In fact all signal-detection theory need do to explain such dissociations is add a new criterion for each additional response category. This amounts however, to adding free parameters. Besides, even if a parsimonious explanation can be found for a given effect, it does not necessarily mean that the explanation is the correct one. Instead of questioning the parsimony of the Dual Process model, the approach taken here has been to challenge the existence of one aspect of this model's support: the relatively small repetition priming effect that has had an influential contribution to our understanding of recognition memory. The results indicate that the effect does not dissociate between subjective experiences of remembering but solely reflects an increase in guessing for primed, but previously unseen, stimuli.

## Competing interests

The author(s) declare that they have no competing interests.

## Authors' contributions

RT designed the studies and made the largest contribution to the manuscript. GF provided much valuable support in conducting and analysing the experiments.
